# Implementation of a Low-Cost Mobile Devices to Support Medical Diagnosis

**DOI:** 10.1155/2013/287089

**Published:** 2013-11-19

**Authors:** Carlos García Sánchez, Guillermo Botella Juan, Fermín Ayuso Márquez, Diego González Rodríguez, Manuel Prieto-Matías, Francisco Tirado Fernández

**Affiliations:** Department of Computer Architecture and Automation, Universidad Complutense de Madrid, 28040 Madrid, Spain

## Abstract

Medical imaging has become an absolutely essential diagnostic tool for clinical practices; at present, pathologies can be detected with an earliness never before known. Its use has not only been relegated to the field of radiology but also, increasingly, to computer-based imaging processes prior to surgery. Motion analysis, in particular, plays an important role in analyzing activities or behaviors of live objects in medicine. This short paper presents several low-cost hardware implementation approaches for the new generation of tablets and/or smartphones for estimating motion compensation and segmentation in medical images. These systems have been optimized for breast cancer diagnosis using magnetic resonance imaging technology with several advantages over traditional X-ray mammography, for example, obtaining patient information during a short period. This paper also addresses the challenge of offering a medical tool that runs on widespread portable devices, both on tablets and/or smartphones to aid in patient diagnostics.

## 1. Introduction

Medical imaging [1] as a diagnostic technique in medicine requires complex image analysis of image sequences obtained by a plethora of variety, such as ECG, X-ray, MRI, ultrasound, CT, and so forth. Magnetic resonance imaging (MRI) [2] technology is one of the most promising tools over other methods, like conventional X-ray mammography, regarding breast cancer diagnosis. Nowadays, X-ray images still have a higher spatial resolution than MR images, but this technique has the advantages of producing natural tridimensional images and being able to noninvasively monitor the contrast agent concentration in the depicted tissue over time.

On other hand, motion estimation is still an open problem with important applications to medical imaging. Attending to the estimation of a pixel motion inside the image sequence, there are many models and algorithms that could be classified as belonging to the matching domain approximations [3], energy models [4], and gradient models [5]. Related to this last family, different studies [6–8] show that this represents an admissible choice for keeping a tolerable trade-off between accuracy and computing resources. For designing systems operating efficiently, many challenges must be dealt with, such as robustness, static patterns, illumination changes, different kinds of noise, contrast invariance, and so forth. 

Regarding the hardware platform used, the portable industry in recent years has dramatically increased the importance of the processing hardware elements. The iPhone 3GS offered more than twice the performance of the first- and second-generation iPhones. As the market becomes more demanding, many processor manufactures have specialized in using smartphones for its own solutions, such as Cortex A8, Snapdragon, ARM11, Tegra, Armada, OMAP, and more. Nowadays, device manufacturers boast about their phones' computer-like capabilities, from desktop-like Internet browsing to HD video playback and, of course, enough computing capability to face up scientific processing [9, 10].

The ARM instruction set has become the reference architecture in low-power devices, so there are many general CPUs able to run compatible ARM code; this fact creates tendency lines in reference processors that fit with these instruction sets, and so the companies produce the chipset following the ARM directives. Some companies, such as Texas Instruments, Samsung, and Nvidia, adopt the ARM CPU reference design, whereas others, like Qualcomm and Marvell, license only the instruction set and create their own processors to fit them.

The popularity of laptops overtook desktop PCs some time ago; mobile devices are currently among the most ubiquitous devices around. Mobile devices are now emerging in two directions: on one hand, they are pushing upward into the territory of Intel Atom-powered netbooks; on the other hand, they are trickling downwards in price, replacing high-end feature phones near the $100 mark.

More than 2 billion ARM chips [11] are shipped every year, beating Intel's Atom devices. The role of GPUs (Graphic Processing Units) is to provide hardware acceleration for tridimensional graphics applications like games, CAD, and so forth. However, in recent years, their role has also become responsible for drawing the main user interface for desktop Operating Systems (OS). On most modern smartphone platforms (iPhone OS, Android, and Palm WebOS, with Windows Mobile as an exception), the OS's user interface itself is composited, meaning it is rendered by the GPU [12]. This makes the interface feel a lot smoother than it would if it were displayed calculations on the already resource-constrained CPU.

Regarding the motion estimation for embedded systems, there are other gradient optical flow models implemented in hardware; some belong to the Lucas and Kanade algorithm [13, 14], and some belong to Horn and Schunk approximations [15, 16]. 

It is worth paying attention to previous implementations [17–19] of the sophisticated and complex Multichannel Gradient Model (McGM) algorithm [20]; this bioinspired algorithm is required to deal with many challenges, such as robustness, static patterns, illumination changes, different kinds of noise, contrast invariance, and so forth. Also the McGM is able to detect correct motion related to optical illusions or avoid operations like matrix inverse or iterative methods that are not biologically justified [15]. We must carefully select a model that carries out these kinds of requirements. There are many implementations for this algorithm [17–19], although we will focus on the Carma board-based [21] implementation. Despite this system's ability to manage complex situations better than others algorithms in real environments [20, 22–25] and mimic some behaviors of mammals [22, 25], its computational complexity is high and still not appropriate for the kind of microprocessors considered.

Under the assumptions mentioned above, in this paper we present a tool to aid medical diagnosis developed *ad hoc* for mobile devices like tablets and/or smartphones. Not only has their acceptance and reduced price driven diffusion but their ever-increasing performance computations offer the opportunity to use them in tasks such as medical diagnosis. This paper studies the feasibility of using this modern technology in a medical scenario, where medical images are processed to aid in medical decisions.

 The present paper is organized as follows. First, the stages of the Lucas and Kanade model and Otsu's method basic equations for segmentation based on histogram are explained very briefly. After that, the basic equations for Otsu's method are explained. Next, the implementation is analyzed using two kinds of microprocessors—an Intel Atom and an ARM processor from the Nvidia Carma board. Several comparisons are performed using existing optical flow implementations in other embedded devices commonly used for mobile platforms. Additionally, output images for the final segmented system are shown. Finally, quality results and associated costs are presented for the hardware.

## 2. Optical Flow Gradient-Based Computation and Segmentation

### 2.1. Optical Flow Estimation and Lucas and Kanade

Assuming that an object moves relative to an imaging device, its 2-dimensional projection usually moves within the projected image. The projection of the 3-dimensional relative motion vectors onto the 2-dimensional detector yields a projected motion field often called the “image flow” or “motion field.” Unfortunately, it is impossible to access the velocity field directly, since optical sensors collect luminance distributions and no speeds. However, it is feasible to compute the motion of local regions of the luminance distribution, and it is this motion field that is referred to as optical flow. The optical flow provides an approximation to the velocity field, but it is uncommonly equal. There are a number of problems to overcome in order to compute the optical flow from the changes in the luminance distribution. First, we can only calculate the motion of patterns, not isolated points, in the luminance distribution. This means that the luminance information must be combined in some way over a finite spatial neighborhood around each point, where we want to measure motion. The so-called aperture problem arises when we try and measure the two components of image velocity using a neighborhood that does not keep enough luminance structure [26–28].

**Figure 1:**
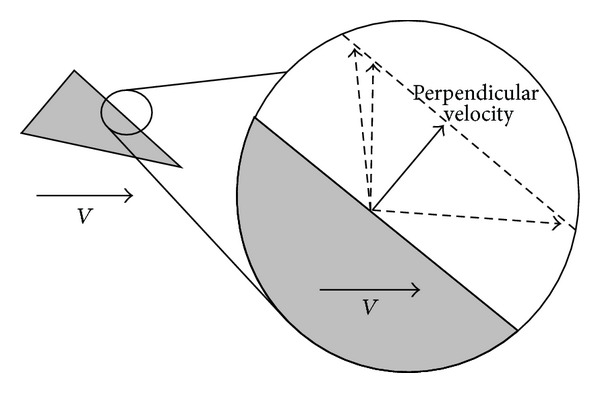
The aperture problem. There are infinite solutions for this problem.

In such situations, we are unable to constrain the measurement to a single solution (Figure 1). Increasing the size of the neighborhood permits us to constrain the measurement, but collecting information over a large region increases the likelihood of pooling over motion boundaries and over smoothing the results, which has been referred to as the general aperture problem [29]. The motion algorithms seek to recover the optical flow field that is the best approximation to the projected velocity field. However, using this information to draw conclusions about the 3-dimensional environment is a difficult process. 

The estimation of the velocity field using optical flow is an ill-posed problem, since there are an infinite number of velocity fields that can cause the observed changes in the luminance distribution. Additionally, there are an infinite number of 3-dimensional motions in the real world that could yield a particular velocity field. External knowledge about the behavior of objects in the real world, such as rigid body motion constraints, are required in order to make use of optical flow. Despite the problems, the optical flow information is a rich array of vectors that has both local and global properties [30]. The optical flow field can thus be subjected to many higher-level interpretations [31, 32]. 

The Lucas and Kanade method [33] is a well-known algorithm, and we have applied the original description of the model [34] while adding several variations to improve the viability of the hardware implementation. We present a simplified scheme of the algorithm, as follows.

The Lucas and Kanade model computes optical flow using a gradient technique [35] that makes use of space-temporal derivative filters. The model comes from the basic intensity conservation over the time (1), where *x*, *y*, and *t* are the coordinates of the sequence. Developing the expression (1), we reach expression (2),

(1) $$\begin{array}{c} \frac{dI\left(x\left(t\right),y\left(t\right),t\right)}{dt}=0, \end{array}$$

(2) dI(x(t),y(t),t)dt =∂I(x(t),y(t),t)∂xdxdt+∂I(x(t),y(t),t)∂ydydt  +∂I(x(t),y(t),t)∂t =∂I(x(t),y(t),t)∂xu+∂I(x(t),y(t),t)∂yv  +∂I(x(t),y(t),t)∂t=0.

The Lucas and Kanade model assumes that the motion vector really does not change in the studied vicinity *V*. Considering the error to minimize the motion constraint expression (2):

(3) E(u,v)=∑pixel∈V(∂I(x(t),y(t),t)∂xu    +∂I(x(t),y(t),t)∂yv    +∂I(x(t),y(t),t)∂t)2.

Solving ∂*E*/∂*u*(*t*) = 0; ∂*E*/∂*v*(*t*) = 0 and grouping them all together, we find an algebraic system expressed by (4), which means the LMS estimation of the optical flow in the centered pixel of the vicinity *V*. The symbol $\hat{\;}$ denotes the estimator of the corresponding magnitude. The resulting optical flow estimated is dense:

(4) [∑pixel∈V∂I(x(t),y(t),t)∂x∂I(x(t),y(t),t)∂x∑pixel∈V∂I(x(t),y(t),t)∂x∂I(x(t),y(t),t)∂y∑pixel∈V∂I(x(t),y(t),t)∂x∂I(x(t),y(t),t)∂y∑pixel∈V∂I(x(t),y(t),t)∂y∂I(x(t),y(t),t)∂y][u^v^] =[−∑pixel∈V∂I(x(t),y(t),t)∂x∂I(x(t),y(t),t)∂t−∑pixel∈V∂I(x(t),y(t),t)∂y∂I(x(t),y(t),t)∂y].

So the final notation is

(5) [u^v^]A=B;A=[∑pixel∈VIx2(pixel)∑pixel∈VIxIy(pixel)∑pixel∈VIxIy(pixel)∑pixel∈VIy2(pixel)]   ×B=[−∑pixel∈VIxIt(pixel)−∑pixel∈VIyIt(pixel)].

The subindex in the equation means the derivatives computed by separable filtering (Gaussian derivatives or Gabor function).

#### 2.1.1. Segmentation by Histogram Using the Otsu Method

The Otsu Method [36, 37] applies an automatic threshold in order to efficiently segment the image; it is based on a discriminant criterion to optimize the function of separation of obtained classes in gray levels. We describe very briefly the method used: if supposing every pixel forms an image represented by gray levels [1,2,…, *L*], the number of the pixels at level *I* is denoted by *n*_*i*_ and the total number of pixels by *N*. The gray-level histogram is normalized regarding a probability distribution expression:

(6) $$\begin{array}{c} p_{i}=\frac{n_{i}}{N},\;\sum_{i}^{L}p_{i}=1. \end{array}$$

Assuming a classification of pixels in two classes *C*_0_ and *C*_1_ (objects and background) by a threshold level *k*, where pixels with levels [1,…*k*] belong to *C*_0_ and pixels with levels [*k* + 1,…, *L*] belongs to *C*_1_, the probabilities of the class occurrence and class means levels are written as

(7) $$\begin{array}{c} w_{0}=\text{Pr}\left(C_{0}\right)=\sum_{i=0}^{k}p_{i};\;\;w_{1}=\text{Pr}\left(C_{1}\right)=\sum_{i=k+1}^{L}p_{i}, \end{array}$$

(8) μ0=∑i=1kiPr(i ∣ C0)=∑i=1kipiwo;μ1=∑i=k+1LiPr(i ∣ C1)=∑i=k+1Lipiw1.

In this step, we are ready to define the following relation for the choice of *k* and the variance based on first-order statistics (class means) as

(9) $$\begin{array}{c} \mu_{T}=w_{o}\mu_{0}+w_{1}\mu_{1};\;\;w_{1}+w_{o}=1;\\ \sigma_{B}^{2}\left(k\right)=\frac{{\left[\mu_{T}w\left(k\right)-\mu \left(k\right)\right]}^{2}}{w\left(k\right)\left[1-w\left(k\right)\right]}. \end{array}$$

The optimal threshold k* that maximizes *σ*_*B*_^2^ is selected by a sequential search using the cumulative quantities expressed in (6) and (7)

(10) σB2(k∗)=max⁡1≤k≤LσB2(k);S∗={k;w0w1=w(k)[1−w(k)]>0}.

## 3. System Implemented

### 3.1. Patients and MR Imaging

Breast MRI was performed on patients with indeterminate mammographic breast lesions. All patients were consecutively selected after clinical examination, mammography in standard projections (craniocaudal and oblique mediolateral projections), and ultrasound. Only lesions classified BIRADS 3 and 4 in mammography were selected. In addition, at least 1 of the following criteria had to be present: nonpalpable lesion, previous surgery with extensive scarring, and location difficult for biopsy (e.g., close to chest wall). Histologic findings were malignant in 14 and benign in 17 lesions. Lesion size was derived from mammography images. Mean size of malignant lesions was 1.2 cm (median = 1.0 cm, range = 0.4–3.5 cm); mean size of benign lesions was 1.1 cm (median = 0.9 cm, range = 0.3−3.0 cm).

MRI was performed with a 1.5 T system (Magnetom Vision, Siemens, Erlangen, Germany) equipped with a dedicated surface coil to enable simultaneous imaging of both breasts. The patients were placed in a prone position. Transversal images were acquired with a STIR (short TI inversion recovery) sequence (TR = 5600 ms, TE = 60 ms, FA = 90°, TI = 150 ms, with a matrix size of 256 × 256 pixels, sliced 4 mm thick). 

Then, a dynamic T1 weighted gradient echo sequence using a 3D FLASH (fast low-angle shot pulse sequence) was performed (TR = 12 ms, TE = 5 ms, FA = 25°) in transversal slice orientation with a matrix size of 256 × 256 pixels and an effective slice thickness of 4 mm. FA (Flip Angle), STIR (Short Tau Inversion Recovery), TE (Echo Time), and TR (Pulse Repetition Interval) are abbreviations for MRI modalities. The dynamic study consisted of 6 measurements with an interval of 83 s. The first frame was acquired before injection of paramagnetic contrast agent (gadopentetate dimeglumine, 0.1 mmol/kg body weight, MagnevistTM, Schering, Berlin, Germany) immediately followed by the 5 other measurements.

### 3.2. Scheme of the System

**Figure 2:**
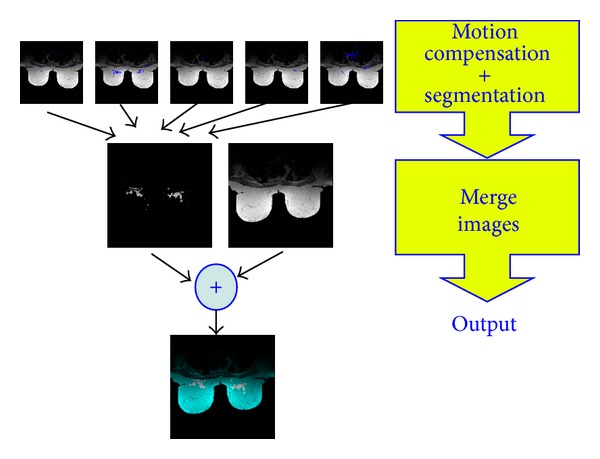
Scheme of the implemented system.

**Figure 3:**
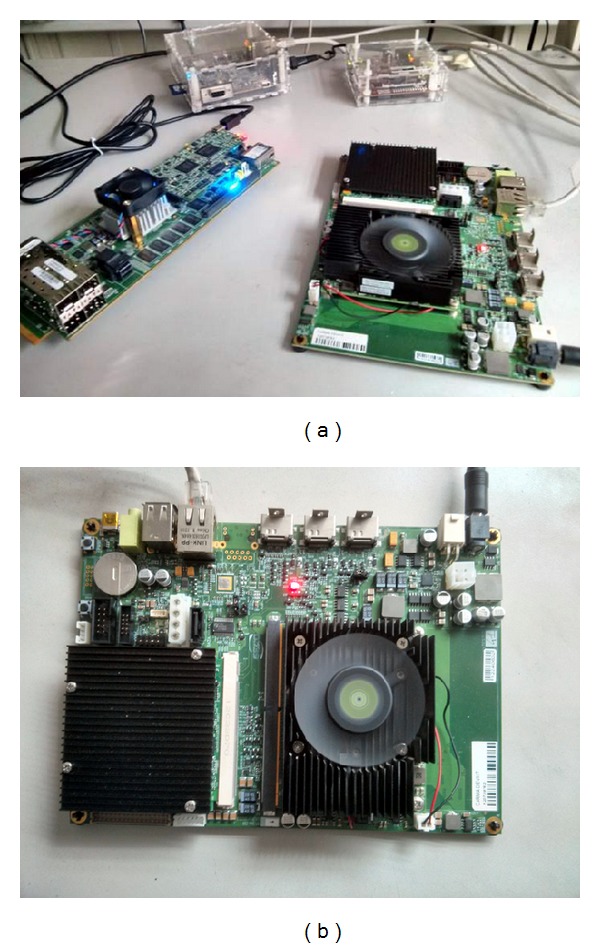
Scheme of the low-cost system implemented at different commercial microprocessors.

Figure 2 shows the algorithm implemented in the embedded system. In the first stage, dense motion is estimated, and the zone corresponding to the range of motion is determined, segmented, and valued using the histogram-based system. It is important to note that this system is adjustable and configurable since the segmented motion values can fit the diagnostic needs, as determined for each individual case. The first system used here was designed using the Carma platform [38] from the SECO company [21], which integrates an Nvidia Tegra CPU with 3 Quad-Core ARM Cortex-A9 CPUs. The second is based on an Intel Atom (2x Intel(R) Atom (TM) CPU D510). In Figure 3, prototyping boards that contain the processors to be programmed are shown.

## 4. Results

In this section, results are shown in the boards based on ARM and ATOM. First we discuss the results in terms of performance (execution time) in both boards and then evaluate the visual results obtained in the proposed medical aid system. We have chosen two systems that incorporate low-power processors which are the base of many mobile devices. Additionally, we have measured how much is affecting the attention windowed motion-zone to be monitored depending on the specific medical diagnostic to be computed.

As we can see in Tables 1 and 2, the performance in terms of seconds/slides (breast cancer stimuli is 256 × 256 and its brain fMRI counterpart is 95 × 69) and final power consumption for both input-stimuli are shown. The results observed demonstrate that system implementation is totally competitive in terms of response time and completely feasible as a tool for medical help.  Table 1 reflects the execution times observed in the sequential application (one processor Atom versus one ARM) and the best configuration obtained in terms of performance (best computing times) corresponding to the exploitation of parallelism with the use of multiple processors. The exploitation of several processors is performed by means of task-level parallelism. Parallelization scheme is based on the uniform distribution of the computational workload among the available processors by means of OpenMP programming paradigm. Accelerations achieved range between 2.2x and 3.3x times faster. For the fMRI brain test, task-level parallelism reports are hardly beneficial; this fact is motivated by the granularity of the problem and the lack of parallelism available to be exploited. The degree of parallelism available in the test considered (lower and middle ranges) makes it unsuitable for the exploitation of additional hardware such as GPU as in the Carma board (the cost of startup, exchange information does not outweigh the benefits that could be achieved in accelerator or GPU). This table also includes a comparison of consumption (peak energy demand) in both systems, so in global terms, we can conclude that a mobile system based on ARM processor reports better performance rates with less power requirements. We would like to highlight that every stage belonging to the system has been designed as customizable, scalable, and modular, containing this system a processing scheme belonging to the most gradient-based optical flow models. As a conclusion, we can affirm that the platforms considered are feasible to process at high scale motion and segmentation attending to the performance obtained at different scale levels.

**Figure 4:**
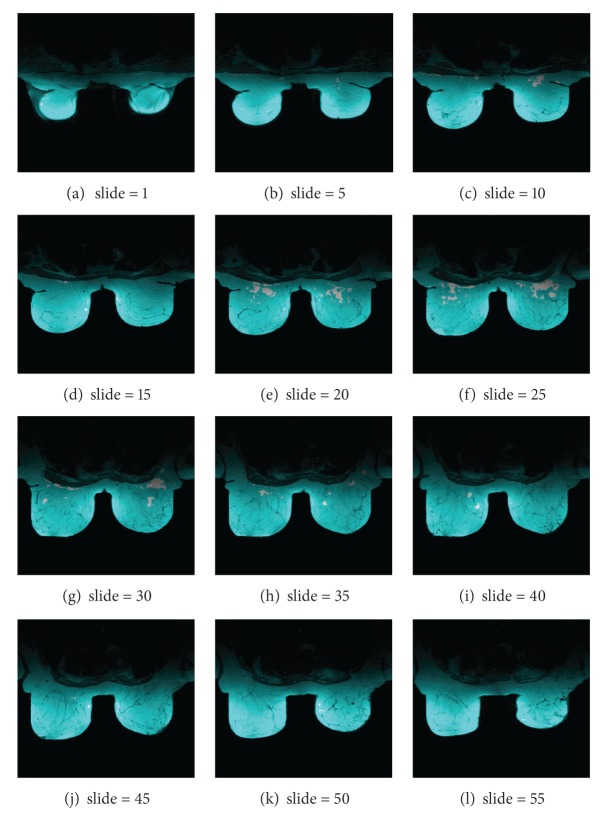
Twelve different slides from the MRI image described in Section 3. The image output from the system is colored for the sake of clarity in recognition. White zones mean high motion density.

**Figure 5:**
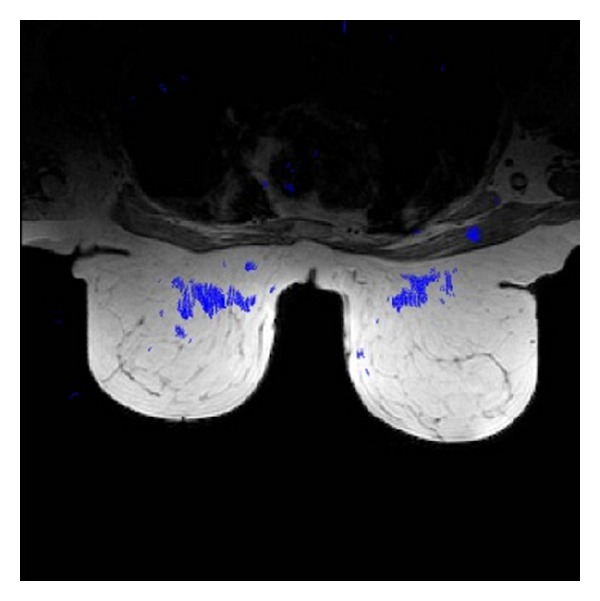
Scheme for motion vector map of one slide.

**Figure 6:**
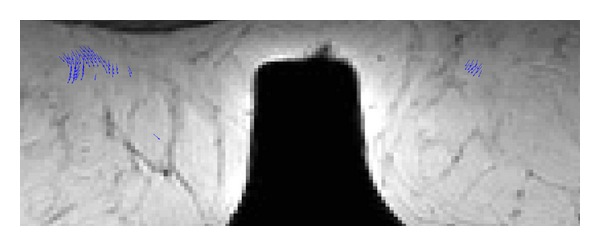
Zoom performed in the output image. Flow vectors corresponding to the adjustable window motion attention zone are shown at different scales in the upper-right and -left part of the image.

**Figure 7:**
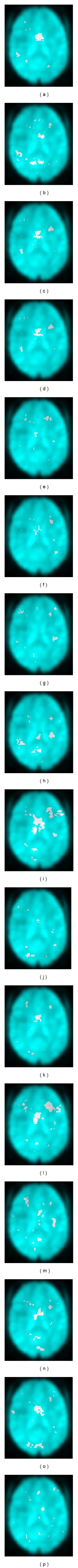
Scheme of the brain image fMRI and motion segmented using the Lucas and Kanade and the Otsu methods.

From the standpoint of the system usefulness and visual results provided, they are also showing some screenshots of the medical analysis generated in a mobile device.  Figure 4 shows a collection of slides from the MRI breast cancer test described in Section 3. The output image displayed on the mobile device is colored for the sake of clarity in recognition, meaning clear-red zones high motion density. Meanwhile Figure 5 illustrates the motion vector map of one slide; Figure 6 addresses a zoom for motion estimation + segmentation output image, where it is possible to recognize the flow vectors corresponding to the adjustable window motion attention zone at different scales in the upper-right and -left part of the image. Additionally, Figure 7 shows a collection of the brain image fMRI test displayed on a tablet where motion segmentation have been performed using the Lucas and Kanade and the Otsu methods.

## 5. Conclusion

This work describes the implementation of a low-cost hybrid system specially designed for mobile devices in medical scenarios where medical images are processed to aid in medical diagnoses and decisions. This system is specifically tuned for breast MRI based on dense motion estimation and segmentation, which can aid specialists in providing rapid attention to breast motion; the present platform can be used as a starting point for motion compensation. The technology can also be utilized for medical diagnosis for remote medicine. These algorithms have been implemented using the same processors as those used in mobile devices, such as tablets, smartphones, and so on.

 Our results have shown that the algorithm is able to detect and visualize motion artifacts with high accuracy. We are currently improving the system with the hierarchical multiscale optical flow algorithm, and we will evaluate the achieved motion correction based on receiver operating characteristic (ROC) over different embedded GPUs in order to export that to mobile devices as well.

## Figures and Tables

**Table 1:** Summary of the final performance (in seconds/slide) for both processors considered and three different motion attention zone selected. (Window) for breast cancer stimuli.

Breast cancer stimuli			
Performance (secs/slide)	ARM v7	Intel ATOM	Final density
Window size = 5			
1 CPU	1,22	0,35	100,00%
Best config.	0,75	0,16	
Window size = 7			
1 CPU	2,12	0,61	100,00%
Best config.	1,18	0,24	
Window size = 9			
1 CPU	3,36	0,92	100,00%
Best config.	1,85	0,28	
Power consumption	8 W	13 W	

**Table 2:** Summary of the final performance (in seconds/slide) for both processors considered and three different motion attention zone selected. (Window) for fMRI Brain Stimuli.

fMRI brain			
Performance (secs/slide)	ARM v7	Intel ATOM	Final density
Window size = 5	0,02	0,02	100,00%
Window size = 7	0,04	0,01	100,00%
Window size = 9	0,06	0,01	100,00%
Power consumption	8 W	13 W	
